# Pharmacokinetics of Two Alkaloids after Oral Administration of *Rhizoma Coptidis* Extract in Normal Rats and Irritable Bowel Syndrome Rats

**DOI:** 10.1155/2014/845048

**Published:** 2014-08-28

**Authors:** Zipeng Gong, Ying Chen, Ruijie Zhang, Yinghan Wang, Qing Yang, Yan Guo, Xiaogang Weng, Shuangrong Gao, Hailin Wang, Xiaoxin Zhu, Yu Dong, Yujie Li, Yajie Wang

**Affiliations:** ^1^Institute of Chinese Materia Medica, China Academy of Chinese Medical Sciences, No. 16, Dongzhimen Nei Nanxiao Road, Dongcheng District, Beijing 100700, China; ^2^Institute of Chinese Materia Medica, Chengde Medical University, Chengde 067000, China; ^3^Guang'an Men Hospital, China Academy of Chinese Medical Sciences, No. 5, Beixiange Road, Xicheng District, Beijing 100053, China

## Abstract

A comparative pharmacokinetic study of berberine and palmatine after oral administration of *Rhizoma Coptidis* extract (96 mg/kg, containing berberine 22 mg/kg and palmatine 5 mg/kg based on body weight) was performed in normal and postinflammation irritable bowel syndrome (PI-IBS) rats, induced by intracolonic instillation of acetic acid and restraint stress. Quantification of berberine and palmatine in rat plasma was achieved by using a sensitive and rapid UPLC-MS/MS method. Plasma samples were collected at 13 different time points and the pharmacokinetic parameters were analyzed by WinNonlin software. The significant differences in the pharmacokinetic behaviors, such as *C*_max⁡_, AUC_(0–*t*)_, *V*_*d*_/*F*, and CL/*F*, of berberine and palmatine were found between normal and PI-IBS model rats. The results indicated that PI-IBS pathological conditions in rats could alter the pharmacokinetic behavior of drug. Preclinical pharmacokinetic studies are usually carried out on healthy animals. However, we should pay more attention to the fact that the change of pharmacokinetic behavior plays an important role on efficacy. It is essential to investigate the pharmacokinetics of the drug in disease status.

## 1. Introduction

Irritable bowel syndrome (IBS) is a chronic functional digestive tract disease, whose symptoms mainly involve not only abdominal pain or distention and abnormal defecation, but also psychological symptoms including anxiety and depression [1]. Postinflammation irritable bowel syndrome (PI-IBS) has been defined as an acute onset IBS symptoms (by Rome criteria) that develop after the individual, who has not previously met the Rome criteria, experiences a gastrointestinal infection with two or more of the following characteristics: fever, vomiting, diarrhea, or a stool culture positive for an infectious agent [2, 3]. However, the pathogenesis of PI-IBS is still unclear, which restrains the development of proper models and drugs for PI-IBS.

*Rhizoma Coptidis* (RC), known as Huang Lian, was officially recognized in the* Chinese Pharmacopoeia *[4] and has been reported to exert a number of pharmacological actions including antispasmodic [5], anti-Alzheimer's disease [6], hypolipidemic [7], anti-inflammatory [8], antitumor [9], and antibacterial [10]. In addition it was described to ameliorate radiation-induced skin injury [11]. Moreover, RC is also used to treat syndromes including abdominal pain and diarrhea [12], including PI-IBS. RC mainly consists of various alkaloids, including berberine, palmatine, coptisine, and epiberberine [13]. Berberine is an important and typical constituent in RC, which possesses a variety of activities including antitumor [14], anti-inflammation [15], and antiatherosclerosis and has also been used to treat infectious diarrhea [16]. The other important constituent of RC is palmatine, which was reported to present some pharmacological effects such as liver-protective and cardiovascular protective effects [17, 18].

In the previous study, we found that the pharmacokinetic profiles of berberine after oral administration of hydrochloride berberine could be significantly altered in PI-IBS rats [19]. Compared with the control group, AUC_(0–*t*)_ significantly increased in the PI-IBS group while CL/*F* considerably decreased. RC is a fundamental herb of traditional Chinese medicine prescriptions used to treat PI-IBS and its side effect is less than the pure berberine. Therefore, in the current study we compared the pharmacokinetics of berberine and palmatine in rat plasma after oral administration of RC extraction in normal and PI-IBS rats using WinNonlin software.

## 2. Materials and Methods

### 2.1. Materials

*Rhizoma Coptidis* is the dried rhizome of* Coptis chinensis* Franch, which was purchased from a local store (Sichuan, China) and identified by Professor Jinda Hao, a botanist and professor at the Institute of Chinese Materia Medica, China Academy of Chinese Medical Sciences. The voucher specimen (no. 110926) has been preserved in our laboratory. Berberine chloride with the purity of 86.7% and palmatine chloride with the purity of 86.1% were obtained from the National Institute for the Control of Pharmaceutical and Biological Products (Beijing, China). Methanol and acetonitrile of chromatographic grade were from Fisher Co., Ltd. (Waltham, USA). Formic acid was obtained from Merck KGaA Co. (Darmstadt, Germany). Deionized water purified by a Milli-Q water purification system (Milford, USA) was used throughout the study. All other reagents were of analytical grade from Beijing Chemical Reagent Co. (Beijing, China).

### 2.2. Preparation of Ethanol Extract of RC

The ethanol extract of RC was prepared by the Department of Pharmacy in China-Japan Friendship Hospital and its main constituents were reported to be alkaloids. The content of berberine and palmatine in the ethanol extract of RC was 23.03% and 5.52% [20].

### 2.3. Animals

Twenty male Sprague-Dawley rats (230–270 g) were obtained from Beijing Vital River Laboratory Animal Technology Co., Ltd. (Beijing, China), housed under standard conditions of temperature, humidity, and light, and had free access to standard rodent diet and water before the experiment. Animal welfare and experimental procedures were strictly in accordance with the Guide for the Care and Use of Laboratory Animals. The animal protocol was approved by the Animal Ethics Committee at the Institute of Chinese Materia Medica, China Academy of Chinese Medical Sciences. Animals were randomly divided into a control and a model group.

### 2.4. Induction of PI-IBS Rats and Administration

After an overnight fast, acute colonic inflammation of 10 rats was induced as PI-IBS model by intracolonic instillation of 4% acetic acid (1 mL) by a silicone tube connected with injector at 8 cm proximal to the anus for 30 s. Then, phosphate buffered saline (1 mL) was instilled to dilute the acetic acid and rinse the colon. The control rats were handled identically except that saline was instilled instead of 4% acetic acid. One week later, the front upper limb, chest, and front porch of PI-IBS model rats were wrapped by adhesive tape for 1 h. The rats had free access to food and water, except when the procedure required deprivation. As previously described [19], distal colonic motility, motility index (MI), number of feces defecated in 2 h, and the time of glass bead output were performed to quantify the visceral hypersensitivity and altered colonic motility after subsidence of inflammation in rats before acetic acid enema and after being crimped.

RC was administered by gastric gavages at the 7 days and behavior tests periods. The control group rats were given distilled water (10 mL/kg).

### 2.5. Drug Analysis

Plasma concentrations of berberine and palmatine were determined by using the UPLC-MS/MS method previously developed and validated [20]. The qualitative chemical profile of these extracts was analyzed by UPLC-MS/MS as previously described [21]. And the content of berberine and palmatine in the ethanol extract of RC was 23.03% and 5.52%.

### 2.6. Pharmacokinetic Analysis

Before the experiment, the rats were fasted overnight and then subjected to the following surgical procedures under anesthesia induced by intraperitoneal injection of chloral hydrate at 150 mg/kg. A polyethylene catheter (0.50 mm i.d., 1.00 mm o.d.; Portex Limited, Hythe, China) was cannulated into the right jugular vein. The distal end of the catheter was led under the skin and exteriorized at the back of the neck. After surgery, the rat was then allowed to recover for 24 h and fasted overnight prior to drug administration. The RC extract freshly dissolves in pure water after dispersion with the aid of an ultrasonic instrument administered intragastrically (i.g.) into rats. After drug administration, the blood samples (200 *μ*L) were collected from the catheter into heparinized centrifuge tubes at appropriate intervals (5 min, 15 min, 30 min, and 1, 1.5, 2, 3, 4, 6, 8, 10, and 12 h). After centrifugation at 3500 rpm for 15 min, 100 *μ*L of plasma was collected and stored at −80°C until analysis. After each blood collection, 200 *μ*L of physiological saline containing 20 units/mL of heparin was immediately injected back into the body to flush the catheter and prevent coagulation. The amounts of berberine and palmatine in plasma were estimated by UPLC-MS/MS analysis as described previously.

### 2.7. Pharmacokinetic Data Analysis

Noncompartmental methods using WinNonlin software (Pharsight Corporation, Mountain View, USA, Version 6.3) were used to analyze plasma concentration* versus* time profiles and estimate the following pharmacokinetic parameters: terminal elimination half-life (*t*_1/2_, *λ*_*z*_); area under the plasma concentration* versus* time curve from zero to the last sampling time (AUC_0–*t*_); volume of distribution (*V*_*d*_, *λ*_*z*_); and total body clearance (CL). The peak plasma concentration (*C*_max⁡_) and the time to reach *C*_max⁡_ (*T*_max⁡_) for i.g. dose were read directly from the observed individual plasma concentration-time data.

### 2.8. Data Analysis

All reported values represent mean ± SD. The statistical difference was calculated using an unpaired* t*-test with a two-tailed distribution for comparison of two mean values. *P* value < 0.05 was considered statistically significant.

## 3. Results

### 3.1. The Change in Body Weight of Rats

**Figure 1:**
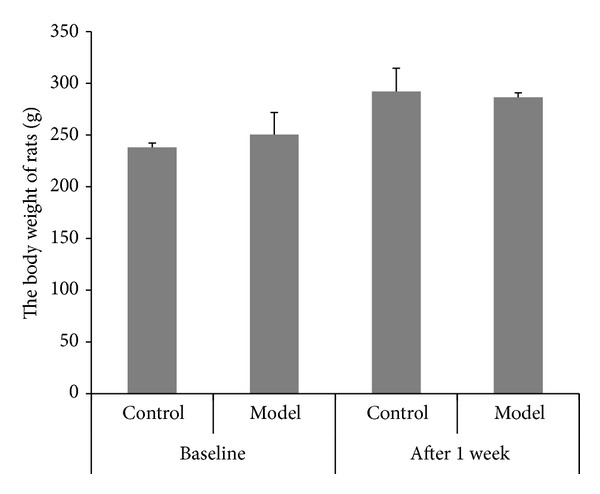
The change of body weight before enema and after stress in rats (mean ± SD, *n* = 10).

Before enema and after stress, the body weights of rats were measured (Figure 1). The increase in body weight of rats in model group was slower than that of rats in control group (238 g* versus* 292 g for control group and 250 g* versus* 286 g for model group). However, the difference between the two groups before enema and after stress was not significant.

### 3.2. Recording of Distal Colonic Motility and Calculation of Motility Index (MI)

**Figure 2:**
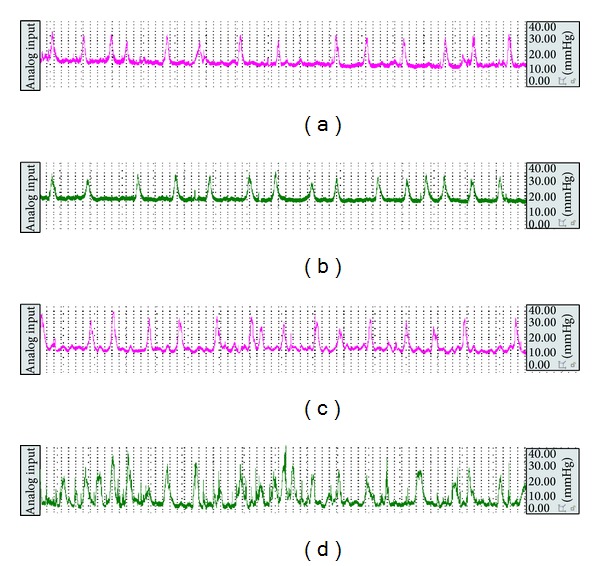
The representative curve of colonic movement in the control group (a) and the model group (b) before enema and in the control group (c) and the model group (d) after stress.

**Figure 3:**
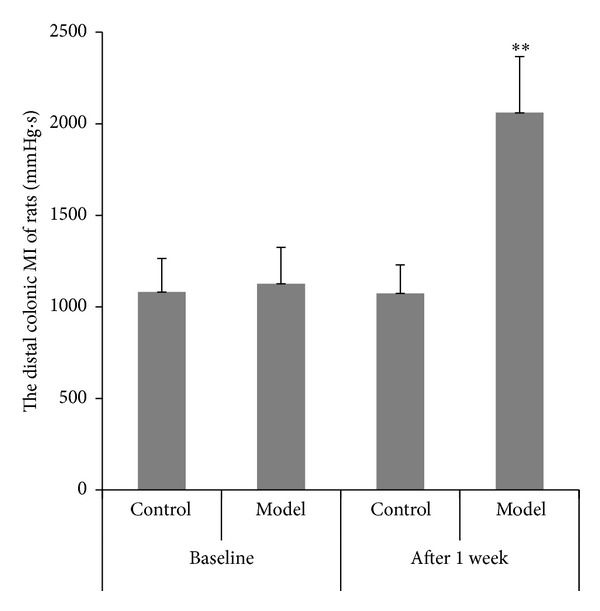
The distal colonic MI of rats in the control group and the model group (mean ± SD, *n* = 10). **P < 0.01 compared with normal group.

Before enema and after stress, the distal colonic MI was observed (Figure 2) and calculated (Figure 3). At the time point before enema, there was no remarkable difference in MI between two groups. After given the stress, the distal colonic MI in the model group was significantly accelerated from 1126.83 mmHg*·*s to 2060.67 mmHg*·*s compared with the control group.

### 3.3. The Time of the Glass Bead Output and the Number of the Fecal Pellet Outputs in 2 Hours

**Figure 4:**
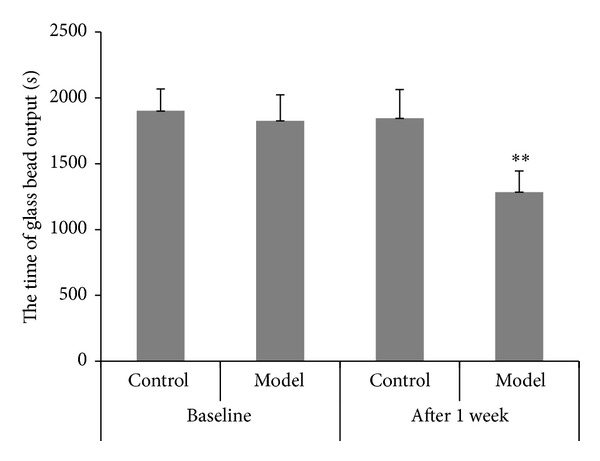
Time of the glass bead output (s) (mean ± SD, *n* = 10). **P < 0.01 compared with normal group.

**Figure 5:**
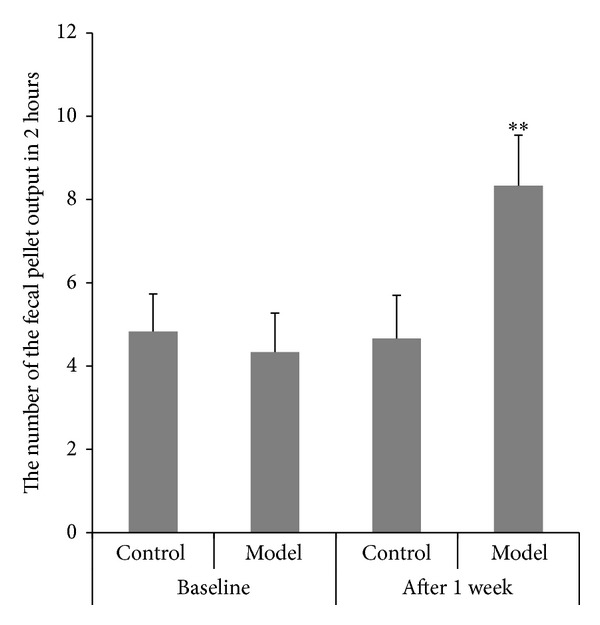
Number of the fecal pellet outputs in 2 hours (mean ± SD, *n* = 10). **P < 0.01 compared with normal group.

Before enema and after stress, the time of the glass bead output (Figure 4) and the number of the fecal pellet outputs in 2 hours (Figure 5) were observed and calculated. At the time point before enema, there was no remarkable difference in the time of the glass bead output and the number of the fecal pellet outputs in 2 hours between the two groups. However, the time of the glass bead output in the model group was significantly shortened and the number of the fecal pellet outputs in model group was significantly increased compared with the control group after given the stress.

### 3.4. Histological Features of Colonic Tissue

**Figure 6:**
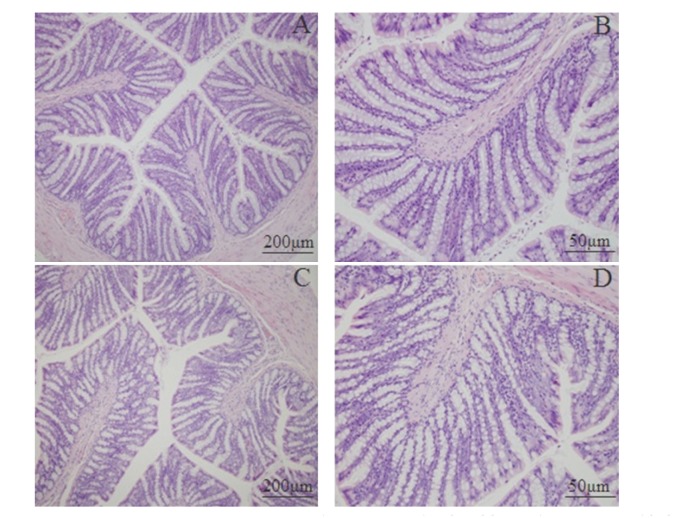
Photomicrographs of distal colons from the control group (A, ×100; B, ×400) and model group (C, ×100; D, ×400) by hematoxylin and eosin staining.

Mucosal histological features in the lamina propria and the submucosa were observed by microscopy.  Figure 6 shows a clear and integral structure of colonic mucosa, including a continuous and integral intestinal epithelium, regular glandular arrangement, and no abnormal cells. In addition, few inflammatory cell infiltrations are seen in the lamina propria. There is no remarkable inflammatory feature in the colon of the rats in the control as well as the model group.

### 3.5. Mast Cells Count and Degranulation Rate in the Proximal Colon

**Figure 7:**
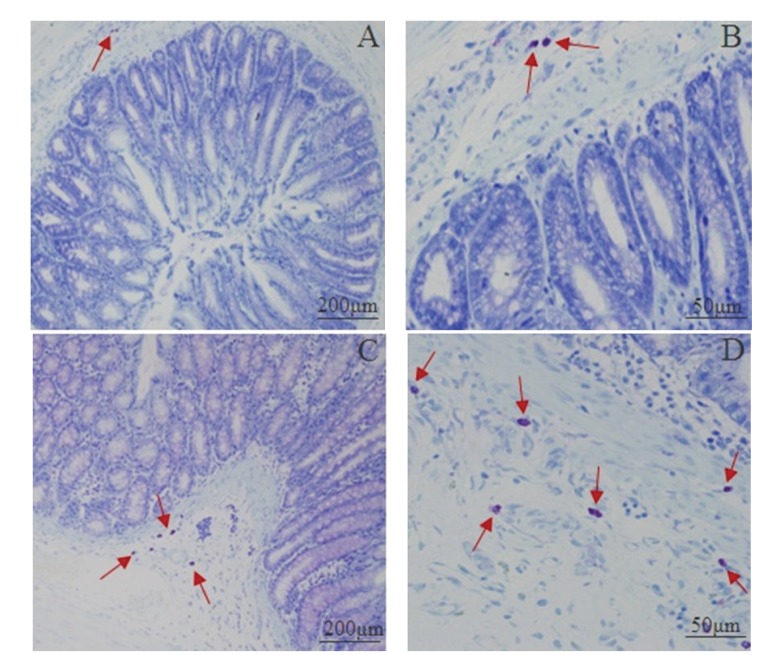
Photomicrographs of mast cell in proximal colons from the control group (A, ×100; B, ×400) and the model group (C, ×100; D, ×400) by toluidine blue staining. The red arrows indicated the mast cell.

Figure 7 shows the distribution and quantity of the mast cells. Most of the mast cells were distributed in the submucosa and lamina propria, in line or around the digestive tract's vessels, lymphatic vessels, and peripheral nerves. Mast cells were round, oval, or irregular, featured as aubergine cytoplasm and blue karyon using a toluidine blue stain. Moreover, the smaller cells had less cytoplasm and clear periphery while the bigger ones had not only more cytoplasm and unclear peripheries but also aubergine granules around the karyon. The distribution of the mast cells in the model group was the same as the control group. However the number of mast cells in the model group was remarkably increased (Table 1). Also the cytomembrane of mast cells in the model group was ruptured and aubergine granules were dispersed in intercellular matrix. These results indicate that intracolonic instillation of acetic acid with restraint stress can cause the anomaly of mast cells.

### 3.6. Pharmacokinetic Analysis

**Figure 8:**
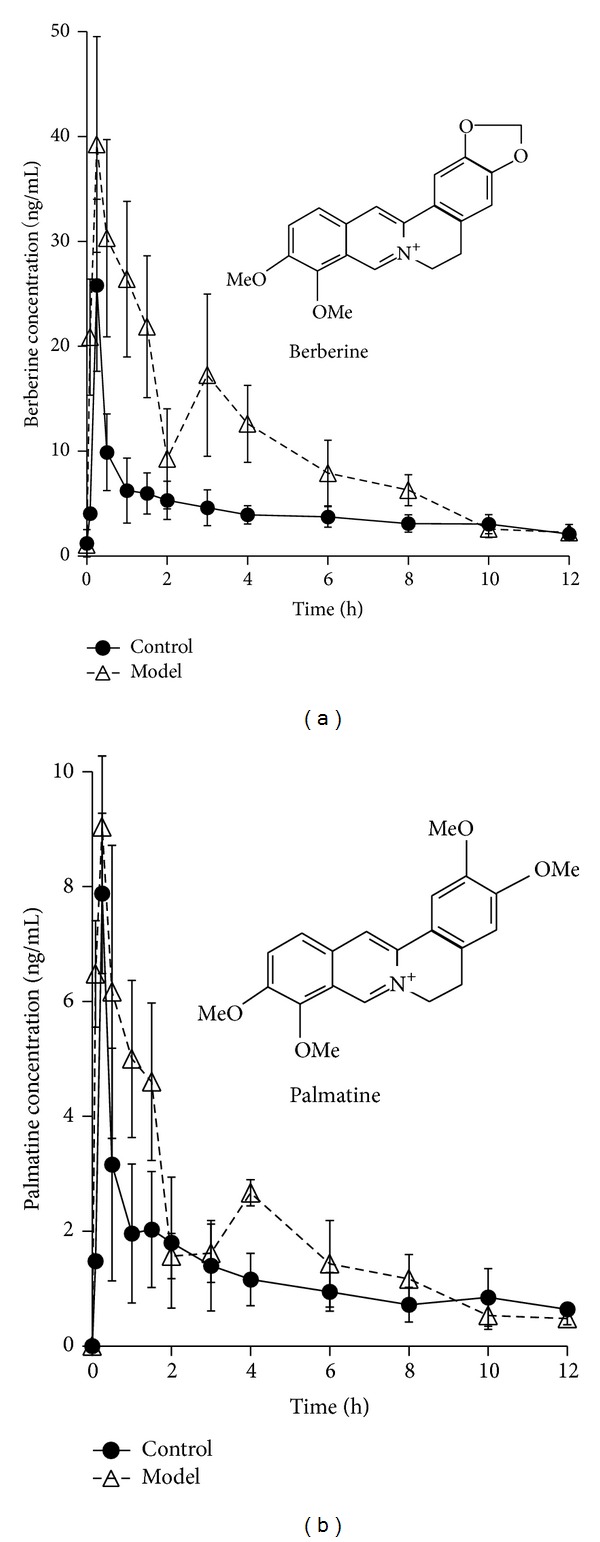
The mean plasma concentration (ng/mL) of berberine and palmatine* versus* time (h) profiles after oral administration of* Rhizoma Coptidis* extract in the control and PI-IBS model rats. Values were expressed as mean ± SD (*n* = 5).

The mean plasma concentration-time profiles of berberine and palmatine following intragastric (i.g.) administration of the RC extract are represented in Figure 8. Also, their pharmacokinetic parameters are summarized in Table 2. The results show that both berberine and palmatine were absorbed rapidly by the body 15 min after intragastric administration of RC extraction in both the control and model groups. Moreover, it is noteworthy that the *C*_max⁡_ and the area under the plasma drug concentration* versus* time curves of berberine were significantly increased in the model group (39.18 ± 7.85 ng/mL; 9874.67 ± 2713.10 min*·*ng/mL) in comparison to the control group (20.04 ± 12.14 ng/mL; 4954.25 ± 784.34 min*·*ng/mL). On the other hand, compared with that in control group (5124.29 ± 1841.24 L/kg; 198.61 ± 75.35 L/h/kg), the marked decrease of *V*_*d*_/*F* and CL/*F* of berberine in the model group (2503.89 ± 542.41 L/kg; 124.34 ± 27.42 L/h/kg) suggested that the elimination of berberine slowed down. For palmatine, the AUC_(0–*t*)_ in the model group significantly increased (1470.61 ± 229.83 min*·*ng/mL* versus *2186.61 ± 693.35 min*·*ng/mL). Additionally, a second peak at 3 hours for berberine and 4 hours for palmatine was observed in the model group.

## 4. Discussion

The curing PI-IBS effect of herbs has been paid more and more attention because of increasing incidence of PI-IBS and lack of effective drugs. Animal models of PI-IBS play an important role in the screening and evaluation of drugs for PI-IBS patients.

In the present study, PI-IBS rat models were established by intracolonic instillation of acetic acid to induce acute inflammation of colon; after the inflammation resolved, wrap restraint stress was given to the rats. During the process of modeling, the rats in the model groups all have serious diarrhea in the three days after enema; this diarrhea gradually resolved in the following days. The body weight in the model group grew slowly while that in the control group grew faster. MI is significantly increased after given stress in the model group. Moreover, the number of mast cells in model group was dramatically increased. However, there was no remarkable inflammatory feature in the colon of the rats in control and model group. These results indicate that intracolonic instillation of acetic acid with wrap restraint stress can largely imitate the symptoms of human PI-IBS and be used as animal models of PI-IBS [22].

RC is a key component of many traditional Chinese medicine prescriptions used to treat syndromes including inflammation [23] and diarrhea, including PI-IBS. However, up to now, little attention has been paid to the pharmacokinetics of alkaloids in irritable bowel syndrome animals and human. Also, drugs are used to treat diseases and only patients are the ultimate consumers of drugs. It is necessary to study the pharmacokinetics of alkaloids in the pathological state. Therefore, in our study, we compared the pharmacokinetics of berberine and palmatine in rat plasma after oral RC at a dosage of 96 mg/kg between normal and PI-IBS rats. The findings showed that *C*_max⁡_ and AUC_(0–*t*)_ of berberine in the model group significantly increased while *V*_*d*_/*F* and CL/*F* decreased. AUC_(0–*t*)_ of palmatine in the model group significantly increased.

Interestingly, a second peak at 3 hours for berberine and 4 hours for palmatine was observed in model group. However, similar results had not happened in the control group. The difference is probably due to the abnormality of colonic motility in the model group. But the results are inconsistent with Deng and Yan's reports [24, 25]. Both of them observed three peaks in mean plasma concentration curves of berberine, palmatine, and jatrorrhizine after oral administration of the combination of* Coptis chinensis* and Fructus Evodiae or RC extraction in normal rats, which is probably concerned with the distribution, reabsorption, and enterohepatic circulation. And the discrepancy probably is because the pharmacokinetic experiments are so susceptible that the mean plasma concentration-time profiles would often differ when the experimental conditions were changed a little, such as investigator, herbs, animals, and sampling time.

In conclusion, the results indicate that the pharmacokinetics of berberine and palmatine after oral administration of RC extraction in rat plasma were significantly different between normal and PI-IBS rats, which indicates a dosage modification of* Rhizoma Coptidis* in PI-IBS pathological conditions.

## 5. Conclusions

The pharmacokinetic behavior of berberine and palmatine after oral administration of* Rhizoma Coptidis* extract was significantly altered in PI-IBS pathological conditions, which indicate the dosage modification of* Rhizoma Coptidis* in PI-IBS.

## Figures and Tables

**Table 1:** The number of the mast cells in proximal colon (mean ± SD, *n* = 5).

Group	Mast cell count after stress (piece)
Control	3.27 ± 1.05
Model	6.77 ± 2.73∗

*P < 0.05 compared with the control group.

**Table 2:** Pharmacokinetic parameters of berberine and palmatine in rat after oral administration of *Rhizoma Coptidis* extract at a dose of 96 mg/kg (mean ± SD, *n* = 5).

Parameters	Berberine		Palmatine	
	Control	Model	Control	Model
*T*_1/2,*λz*_ (min)	945.8 ± 349.5	836.2 ± 253.3	1611.7 ± 616.0	1521.7 ± 710.9
*T*_max⁡_ (min)	15.0 ± 0.0	15.0 ± 0.0	15.0 ± 0.0	15.0 ± 0.0
*C*_max⁡_ (ng/mL)	20.0 ± 12.1	39.2 ± 7.9∗∗	8.1 ± 2.2	9.1 ± 1.2
AUC_0–t_ (min*·*ng/mL)	4954.3 ± 784.3	9874.7 ± 2713.1∗∗	1470.6 ± 229.8	2186.6 ± 693.4∗
*V*_d_/*F*_*λz*_ (L/kg)	5124.3 ± 1841.2	2503.9 ± 542.4∗∗	4869.6 ± 1237.6	4315.9 ± 1111.3
CL/*F* (L/h/kg)	198.6 ± 75.4	124.3 ± 27.4∗	147.4 ± 45.3	119.3 ± 28.1

**P < 0.01 and ∗*P* < 0.05 compared with the control group.
